# Effect of Strontium Peroxide and Copper-Doped Hydroxyapatite Microceramics on the Osteogenesis and Antibacterial Activity of Nanofibrous Composite Scaffolds

**DOI:** 10.3390/ma19142982

**Published:** 2026-07-10

**Authors:** Pan-Geon Park, Young-Jin Kim

**Affiliations:** Department of Advanced Materials and Chemical Engineering, Daegu Catholic University, Gyeongsan 38430, Republic of Korea; psb7193@naver.com

**Keywords:** antibacterial activity, osteogenesis, strontium peroxide, copper-doped hydroxyapatite, composite scaffold

## Abstract

Engineering multifunctional scaffolds that effectively promote bone regeneration while concurrently mitigating the risk of infection remains a significant challenge in the field of bone tissue engineering. In this study, we present the fabrication of electrospun poly(lactic acid) (PLA) nanofibrous composite scaffold (PLASrCu) incorporating strontium peroxide (SrO_2_) and copper-doped hydroxyapatite (CuHA) particles. The resulting composite scaffold exhibited interconnected porous structures and extracellular matrix-like morphologies. Physicochemical characterization confirmed the preservation of PLA chemical structure and the successful incorporation of crystalline SrO_2_ and CuHA phases, with the tensile strength increasing from 2.3 to 2.8 MPa. The PLASrCu scaffold exhibited sustained ion release of Sr and Cu (12.2 and 13.3 mg/L, respectively, over 14 days), together with controlled oxygen generation (10.2 mg/L within 30 min), particularly under hypoxic conditions. In vitro biological assessments demonstrated that the PLASrCu scaffold significantly enhanced cell proliferation and viability. Moreover, osteogenic differentiation and mineralization were markedly promoted, as evidenced by upregulated expression of COL1, OPN, and RUNX2 (5.1-, 2.6-, and 1.9-fold increases, respectively) and increased calcium deposition. Importantly, the Cu-containing scaffolds effectively inhibited the growth of Staphylococcus aureus and Pseudomonas aeruginosa, resulting in antibacterial rates above 99.9%. Collectively, these results demonstrate that the PLASrCu nanofibrous scaffold integrates osteogenic, oxygen-generating, and antibacterial functions within a single platform, highlighting its strong potential for the regeneration of infected and oxygen-deficient bone defects.

## 1. Introduction

The repair of damaged bone resulting from osteoporosis-related fractures, osteonecrosis, trauma, and tumor resection is achieved through the interplay of bioactive scaffolds, bone-forming progenitor cells, and osteogenic growth factors to promote bone tissue regeneration [1,2]. Vascularized autologous bone grafts represent the current gold standard for the treatment of critical-sized bone defects owing to their excellent regenerative capacity. However, their widespread clinical application is restricted due to donor-site morbidity and the scarcity of available graft material [3,4]. Therefore, tissue-engineered porous scaffolds have been considered as viable synthetic alternatives for autologous bone grafting, as they possess interconnected structures that mimic the natural bone extracellular matrix to support osteogenesis and angiogenesis [5,6].

Oxygen plays indispensable metabolic and regulatory roles by supporting cellular metabolism and modulating key cellular behaviors [5]. Accordingly, hypoxic conditions arising from vascular disruption after bone fracture adversely affect bone healing by inducing cell death and inhibiting osteoblast differentiation [7]. Although tissue-engineered scaffolds are excellent candidates for bone graft substitution, the limited diffusion of oxygen into non-vascularized regions of bone defects prior to neovascularization can result in an insufficient oxygen supply to cells, creating a hypoxic microenvironment that promotes osteonecrosis [8]. To address this issue, several oxygen-delivery strategies, including hyperbaric oxygen therapy, oxygen carriers, and peroxide-based materials, have been explored for the treatment of bone defects [5,6,7,8,9]. Hyperbaric oxygen therapy is clinically available and increases tissue oxygenation; however, it cannot provide sustained oxygen delivery to bone defects due to restricted oxygen diffusion [10]. Therefore, engineered bone grafts capable of sustained oxygen release are needed to enhance local oxygen availability and support efficient bone repair.

To date, various peroxide-based materials, including strontium peroxide (SrO_2_) and calcium peroxide, have been engineered to restore local oxygen homeostasis and preserve cellular viability under hypoxia [7,10,11]. These materials undergo hydrolysis to form hydrogen peroxide (H_2_O_2_), followed by its decomposition into oxygen and water. Since the kinetics of oxygen generation are highly dependent on environmental parameters, including pH and catalyst availability, peroxide-based materials have been widely integrated into various biomaterials to achieve sustained oxygen release [7,10]. However, despite the progress made, peroxide-based oxygen-delivery systems still face challenges, including slow and uncontrolled bursts of oxygen release [7,12]. Therefore, their ability to simultaneously promote bone regeneration under hypoxic conditions and inhibit microbial infection remains insufficiently explored. Therefore, the development of advanced oxygen-generating platforms remains a significant challenge.

Biomaterials with intrinsic antibacterial activity are highly sought in a wide range of clinical applications. In particular, bone graft substitutes are frequently required in challenging trials, such as open fractures accompanied by substantial bone loss [13,14]. Under these conditions, extensive wound contamination often leads to tissue infection and osteomyelitis, which severely impair new bone formation and compromise the healing process. Moreover, the widespread use of broad-spectrum antibiotics has markedly exacerbated the emergence of multidrug-resistant bacterial strains in patients with second infections [13]. Such infections are associated with significantly poorer clinical outcomes compared with cases without co-infection. Therefore, the development of innovative bone substitute materials with inherent antibacterial properties is essential for reducing or eliminating excessive antibiotic usage.

Bioactive metal oxides and bioceramics have recently been incorporated into various natural and synthetic polymers to fabricate nanofibrous biomimetic scaffolds [13,14,15,16]. These scaffolds offer interconnected pore structures, adequate mechanical properties, and controlled release characteristics, thereby enhancing osteogenesis and angiogenesis. Moreover, they enable precise geometric conformity to irregular bone defects while creating interconnected three-dimensional architectures that promote nutrient diffusion and cell proliferation.

SrO_2_ particles have been incorporated into biomaterials as bioactive cues to enhance osteogenic activity and inhibit osteoclastic resorption [17]. Their mechanism involves stimulation of osteogenic gene expression, osteoprotegerin (OPG) secretion, and alkaline phosphatase activity in osteoblastic cells. In addition, owing to transport mechanisms analogous to those of Ca ions, Sr ions are readily incorporated into the bone matrix during the mineralization process [18]. However, SrO_2_ particles suffer from several drawbacks, including slow and uncontrolled oxygen generation and a lack of antibacterial activity. Hydroxyapatite (HA)-based bioceramics have been widely used clinically for bone defect repair [19]. HA exhibits excellent biocompatibility and bioactivity owing to its chemical similarity to native bone mineral. In particular, metal-ion-doped HAs (e.g., Cu^2+^- and Sr^2+^-doped HA) show sustained ion release, enhanced apatite formation, and increased cell proliferation, thereby promoting bone tissue regeneration [20,21]. However, despite their favorable biological properties, metal-ion-doped HAs lack oxygen-generating capability. Therefore, there is a growing need to develop multifunctional platforms that simultaneously provide oxygen, inhibit microbial infection, and promote bone tissue regeneration under hypoxic conditions.

Herein, we introduce a novel fabrication strategy for multifunctional nanofibrous composite scaffolds comprising SrO_2_ and Cu-doped HA embedded in a polylactic acid (PLA) as advanced metal ion- and oxygen-delivery platforms via electrospinning. The resulting nanofibrous scaffolds demonstrated improved oxygen-generating capacity, osteoconductivity, and antibacterial activity. A systematic investigation was conducted to elucidate the effects of SrO_2_ and Cu-doped HA (CuHA) incorporation on the physicochemical properties, controlled oxygen-release behavior, and biological performance of the composite scaffolds, thereby evaluating their potential as bone regenerative biomaterials.

## 2. Materials and Methods

### 2.1. Materials

PLA (Ingeo^TM^ Biopolymer 6362D, Mw = 1.6 × 10^5^ g mol^−1^) was obtained from NatureWorks LLC (Minnetonka, MN, USA). Calcium nitrate tetrahydrate (Ca(NO_3_)_2_·4H_2_O, Assay 99%), ammonium phosphate dibasic ((NH_4_)_2_HPO_4_, Assay ≥ 98%), copper(II) nitrate trihydrate (Cu(NO_3_)_2_·3H_2_O, Assay ≥ 98%), strontium chloride hyxahydrate (SrCl_2_·6H_2_O, Assay ≥ 98%), ammonium hydroxide solution (25 *w*/*v*%, NH_3_·H_2_O), hydrogen peroxide (H_2_O_2_, 30 *w*/*w*%), poly(ethylene glycol)-block-poly(propylene glycol)-block-poly(ethylene glycol) (PEG-PPG-PEG, *M*_n_ = 8400), sodium alginate, hyaluronic acid sodium salt from *Streptococcus equi* (8–15 kDa), alizarin red S (ARS), dichloromethane, 3-(4,5-dimethylthiazol-2-yl)-2,5-diphenyltetrazoliumbromide (MTT), and N,N-dimethylformamide (DMF, Assay ≥ 99.8%) were purchased from Sigma-Aldrich Co. (St. Louis, MO, USA).

Mouse calvaria preosteoblast cell line (MC3T3-E1, CRL-2593™) was purchased from the American Type Culture Collection (ATCC, Manassas, VA, USA). Fetal bovine serum (FBS), minimum essential medium alpha (MEM-α), Dulbecco’s phosphate-buffered saline (DPBS, pH 7.4), and penicillin–streptomycin were obtained from Gibco BRL (Waltham, MA, USA). The LIVE/DEAD Viability/Cytotoxicity Assay Kit was purchased from Molecular Probes (Eugene, OR, USA), and the Actin Cytoskeleton and Focal Adhesion Staining Kit was obtained from Merck Millipore (Burlington, MA, USA).

### 2.2. Synthesis of CuHA Particles

CuHA particles were synthesized as follows. A 0.1 M (NH_4_)_2_HPO_4_ solution (pH 10) was added over 2 h to mixed aqueous solutions (pH 10) of 0.1 M Cu(NO_3_)_2_·3H_2_O and 0.1 M Ca(NO_3_)_2_·4H_2_O containing 0.03 *w*/*v*% sodium alginate, keeping the (Ca + Cu)/P ratio at 1.67. The Cu/(Ca + Cu) molar ratio in the reactant solution was 0.3. The suspension was subjected to vigorous stirring at 45 °C for 24 h, thereby promoting the nucleation and crystal growth of CuHA particles. The resulting particles were collected by repeated rinsing with deionized water (DW), lyophilized, and thermally treated at 800 °C for 2 h. Particle size distribution analysis of the CuHA particles was carried out using dynamic light scattering (DLS) on a SALD-7500nano (Shimadzu, Kyoto, Japan).

### 2.3. Synthesis of SrO_2_ Particles

SrO_2_ particles were synthesized by dissolving SrCl_2_·6H_2_O (3.0 g) in 60 mL of aqueous solution containing 6.0 g of PEG-PPG-PEG and 0.06 g of hyaluronic acid sodium salt. Following pH adjustment of the reaction solution to 10 using NH_3_·H_2_O solution, the mixture was stirred for 1 h. Subsequently, 15 mL of 30 wt% H_2_O_2_ was slowly added dropwise under continuous stirring, followed by vigorous stirring at 40 °C for 3 h to induce the nucleation and growth of SrO_2_ particles. The synthesized particles were purified by repeated washing with ethanol and vacuum-dried at 65 °C.

### 2.4. Fabrication of PLA Nanofibrous Composite Scaffolds

Before electrospinning, PLA (2.0 g) was dissolved in 10 mL of a mixed solvent of dichloromethane/DMF (3:2 *v*/*v*) to obtain a 20 *w*/*w*% solution and stirred for 12 h. SrO_2_ (0.2 g) and/or CuHA (0.8 g) particles were then added and dispersed at 40 °C for 6 h. The precursor was loaded into a 20 mL syringe equipped with a 22 -gauge stainless steel needle (internal diameter = 0.41 mm). Nanofibers were fabricated by electrospinning at room temperature under a feed rate of 1 mL/h, an applied voltage of 18 kV, and a working distance of 12 cm. The PLA nanofibrous composite scaffolds were peeled off from the stainless steel collector and vacuum-dried at 40 °C for 24 h.

### 2.5. Surface Morphology and Physicochemical Properties of PLA Nanofibrous Composite Scaffolds

The morphology and microstructure of the PLA nanofibrous composite scaffolds were characterized by scanning electronic microscope (SEM; Mira III, TESCAN, Brno, Czech Republic), and their elemental composition and distribution were subsequently analyzed by energy-dispersive X-ray spectroscopy (EDX) coupled with SEM. The chemical composition of the composite scaffolds was further identified by attenuated total reflectance–Fourier transform infrared spectroscopy (ATR–FTIR; ALPHA spectrometer, Bruker Optics, Billerica, MA, USA). The crystalline structure of the composite scaffolds was characterized by X-ray diffraction (XRD; Empyrean, Malvern Panalytical, Malvern, UK) using CuKα radiation. Mechanical testing was performed using a universal testing machine (AGS-X, Shimadzu, Kyoto, Japan) at a crosshead speed of 1 mm/min, with measurements obtained from five independent specimens in each group.

### 2.6. Oxygen Generation and Ion Release Assays

Oxygen generation from the composite scaffolds was assessed by measuring the dissolved oxygen concentration in the aqueous medium after scaffold immersion (15 mg/mL). Oxygen concentration was monitored for 30 min at room temperature using an oxygen dissolving meter (HI-9147, Hanna Instruments, Woonsocket, RI, USA). The cumulative release of Sr and Cu ions from the PLASrCu scaffold was determined quantitatively over a 14-day period using inductively coupled plasma mass spectrometry (ICP-MS; Agilent 7800 Quadrupole, Agilent Technologies Inc., Santa Clara, CA, USA). Briefly, composite scaffolds (50 mg) were immersed in 5 mL of DW and maintained in a temperature-controlled shaker at 37 °C with agitation at 100 rpm. At selected time points throughout the incubation period, the supernatants were collected for analysis. The cumulative concentrations of released Sr and Cu ions were subsequently quantified, and their time-dependent release behaviors were analyzed.

### 2.7. Cell Proliferation on the PLA Nanofibrous Composite Scaffolds

The biological activity of the PLA nanofibrous composite scaffolds was assessed using MC3T3-E1 preosteoblasts (ATCC, CRL-2593™) [22]. Cells were maintained in MEM-α containing 10% FBS and 1% penicillin–streptomycin under standard culture conditions (37 °C, 5% CO_2_). To evaluate cell growth under hypoxia, cells were cultured under hypoxic conditions (1% O_2_). The scaffolds were sterilized by sequential immersion in 75%, 50%, and 25% ethanol, followed by four washes with DPBS and MEM-α, and placed in 24-well tissue culture plates using glass rings for fixation. MC3T3-E1 cells were seeded onto each composite scaffold at a density of 5 × 10^4^ cells per well and maintained for 5 and 10 days. Cell proliferation was quantified with the MTT assay, and cell attachment on the scaffolds was observed by SEM. After 5 and 10 days of culture, cells were fixed in 4% paraformaldehyde, washed with DPBS, dehydrated with ethanol, vacuum-dried, and platinum-coated for SEM imaging.

Cell viability on the composite scaffolds was assessed using a live/dead staining assay, in which live cells were labeled green by calcein acetoxymethyl ester (calcein AM) [23]. MC3T3-E1 cells were seeded onto each composite scaffold at a density of 5 × 10^4^ cells per well. After culturing for 5 days, the MC3T3-E1 cells were incubated with 1 μM calcein AM for 30 min at room temperature, washed with DPBS, post-incubated for 24 h, and imaged using a confocal laser scanning microscope (LSM 700, Carl Zeiss, Oberkochen, Germany).

For cytoskeletal visualization, MC3T3-E1 cells were seeded onto the composite scaffolds at a density of 5 × 10^4^ cells per well and maintained for 5 days under hypoxia. Cells were fixed, permeabilized with 0.1 *w*/*v*% Triton-X 100 for 5 min, blocked with 1 wt% BSA, stained with phalloidin-TRITC and DAPI, and imaged using a confocal laser scanning microscope.

### 2.8. Cell Differentiation and Mineralization on the PLA Nanofibrous Composite Scaffolds

To evaluate osteogenic differentiation, the expression levels of osteogenic marker genes (collagen type 1 (COL1), runt-related gene 2 (RUNX2), and osteopontin (OPN)) in MC3T3-E1 cell culture on the PLA nanofibrous composite scaffolds were quantified by quantitative real-time reverse transcription polymerase chain reaction (qRT-PCR). MC3T3-E1 cells were seeded onto each composite scaffold at a density of 5 × 10^4^ cells per well and maintained under hypoxia for 14 days. Total RNA was isolated using TRI-Solution™ (BSK Bio, Daegu, Republic of Korea). First-strand cDNA was synthesized using the PrimeScript™ RT Reagent Kit (Takara, Shiga, Japan). Quantitative real-time PCR was performed with gene-specific primers using the GoTaq^®^ qPCR and RT-qPCR Systems (Promega, Madison, WI, USA). Relative gene expression levels were normalized to the housekeeping gene glyceraldehyde-3-phosphate dehydrogenase (GAPDH).

The calcium deposition capability of MC3T3-E1cells cultured on the composite scaffolds was evaluated using Alizarin Red S (ARS) staining, as ARS selectively binds to calcium ions [2,14]. MC3T3-E1 cells were seeded onto the composite scaffolds at a density of 5 × 10^4^ cells per well and cultured for 14 days prior to fixation with 4% glutaraldehyde. The samples were then stained with 1 mL of ARS solution (40 mM, pH 4.2) for 30 min. After staining, the samples were rinsed three times with DW, and calcium nodule formation was visualized using a microscope (Eclipse TS100, Nikon, Tokyo, Japan).

### 2.9. Antibacterial Activity Assay

The antibacterial performance of the composite scaffolds was assessed against the Gram-positive bacterium *Staphylococcus aureus* (*S. aureus*, ATCC 29213) and the Gram-negative bacterium *Pseudomonas aeruginosa* (*P. aeruginosa*, ATCC 9027). These bacterial strains were obtained from the Korea Culture Center of Microorganisms (Seoul, Republic of Korea). The composite scaffolds (50 mg/mL) were sterilized and preincubated in 4.5 mL of saline at 37 °C for 72 h. Then, 0.5 mL of 100 CFU/mL bacteria was added and maintained at 37 °C for 24 h. After incubation, the bacterial suspensions were vortexed to detach adherent bacteria, serially diluted 1000-fold, and plated (0.1 mL per plate) on Tryptic Soy Agar. The plates were incubated for 24 h, after which bacterial colonies were imaged.

### 2.10. Statistical Analysis

All quantitative data are expressed as the mean ± standard deviation (SD). All experiments were independently performed at least five times. Statistical analysis was conducted using one-way analysis of variance (ANOVA), followed by Tukey’s multiple comparison test, with SigmaPlot 13.0 software (Systat Software Inc., San Jose, CA, USA). Differences were considered statistically significant at *p* < 0.05.

## 3. Results and Discussion

### 3.1. Fabrication of PLA Nanofibrous Composite Scaffolds

One of the most promising strategies for designing advanced biomaterials for bone defect repair is the integration of multiple functionalities into a single system, including osteoconductive properties and the capability of promoting osteogenic differentiation to guide new bone formation [24]. However, bone healing is often severely compromised in the presence of infection, particularly following open fractures. Conventional treatments, such as systematic antibiotic administration and surgical debridement, may lead to serious complications and frequently require additional surgical interventions. As a result, the design of bioactive materials with intrinsic antibacterial activity has emerged as an effective strategy for preventing postoperative infections.

It has been reported that the release of Sr and Cu ions from bioactive materials can significantly enhance bone tissue regeneration by promoting osteogenesis, angiogenesis, and antibacterial activity, thereby facilitating bone defect repair [14,20,21]. In addition, efficient intracellular oxygen generation within non-vascularized regions of bone defects has been shown to improve local tissue oxygenation and accelerate bone healing. Accordingly, for effective bone regeneration, an ideal scaffold is expected to possess osteoconductivity, oxygen-generating capability, antibacterial activity, superior biocompatibility, and a highly porous structure [1,5,14].

**Figure 1:**
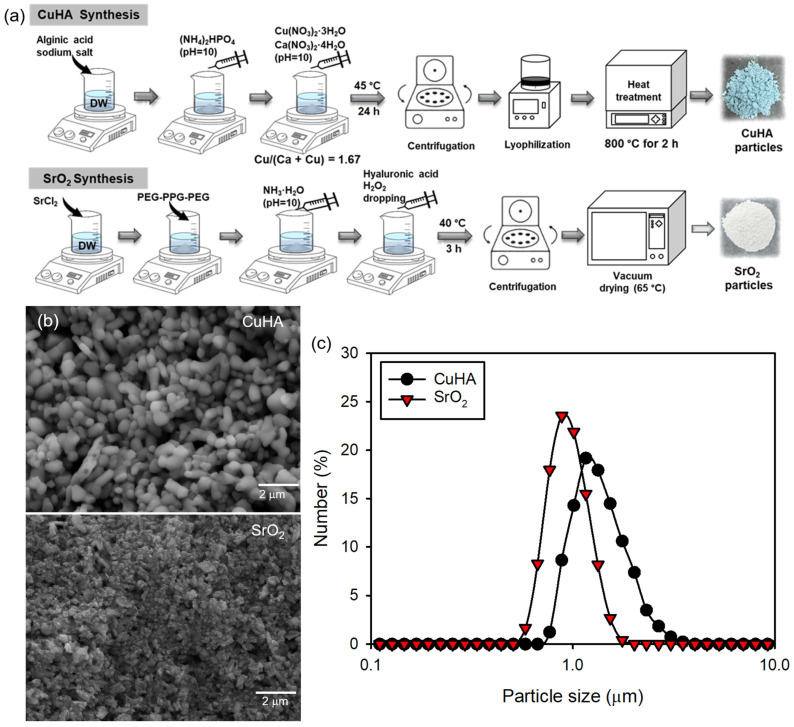
(**a**) Schematic diagram of the synthetic process of CuHA and SrO_2_ particles. (**b**) SEM images and (**c**) particle size distribution of CuHA and SrO_2_ particles.

In the present study, electrospun PLA nanofibrous composite scaffolds were fabricated by incorporating synthesized CuHA and SrO_2_ particles. As shown in the SEM images in Figure 1a, both CuHA and SrO_2_ particles exhibited a quasi-spherical morphology with submicron dimensions. Even though the particles were within the submicron size, noticeable aggregation among particles was observed, resulting in average aggregate sizes of 1428 ± 189 nm for CuHA and 991 ± 135 nm for SrO_2_ (Figure 1b).

**Figure 2:**
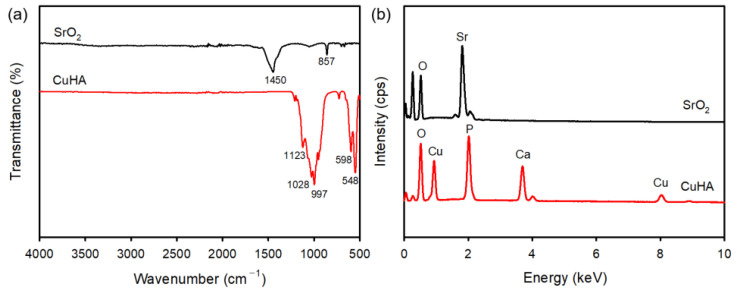
(**a**) FTIR and (**b**) EDX spectra of CuHA and SrO_2_ particles.

The chemical structures of the synthesized particles were confirmed using FTIR spectroscopy (Figure 2a). CuHA particles displayed characteristic peaks at 1123, 1028, and 997 cm^−1^ associated with the P–O stretching modes of phosphate groups in HA [20], while characteristic bands at 598 and 548 cm^−1^ were assigned to O–P–O bending modes. No noticeable changes in absorption bands were observed upon Cu doping in HA. In contrast, SrO_2_ particles exhibited absorption bands at 1450 and 857 cm^−1^, which were assigned to the Sr–O–Sr and Sr=O stretching modes, respectively [2]. The elemental compositions of the particles were further verified by EDX analysis, which revealed the presence of O, Cu, P, and Ca in CuHA particles and O and Sr in SrO_2_ particles (Figure 2b).

**Figure 3:**
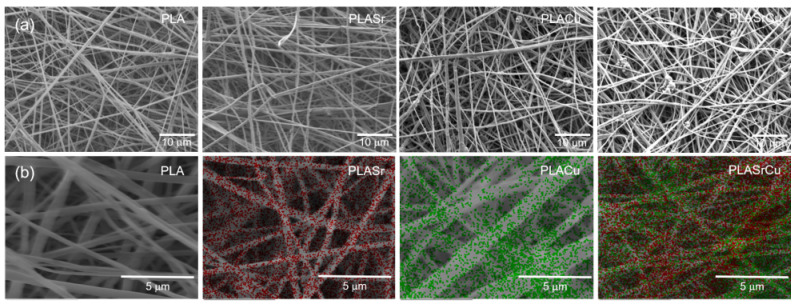
(**a**) SEM images and (**b**) EDX mapping (Sr: red; Cu: green) of the composite scaffolds.

PLA nanofibrous composite scaffolds were subsequently fabricated by electrospinning PLA solutions containing CuHA and/or SrO_2_ particles, yielding PLASr (PLA/SrO_2_), PLACu (PLA/CuHA), and PLASrCu (PLA/SrO_2_/CuHA) scaffolds. A pristine PLA scaffold without particles served as the control. SEM images demonstrated that all composite scaffolds possessed interconnected pore structures and extracellular matrix (ECM)-like architectures (Figure 3a). The fiber diameters were 888 ± 183 nm for PLA, 871 ± 140 nm for PLASr, 829 ± 147 nm for PLACu, and 836 ± 136 nm for PLASrCu. The slight reduction in fiber diameter for the composite scaffolds was attributed to bead formation induced by the particle incorporation. In addition, EDX elemental mapping demonstrated the homogeneous distribution of CuHA and SrO_2_ within the composite scaffolds, with Sr and Cu signals clearly detected in the PLASrCu scaffold (Figure 3b).

### 3.2. Physicochemical Characterization of PLA Nanofibrous Composite Scaffolds

**Figure 4:**
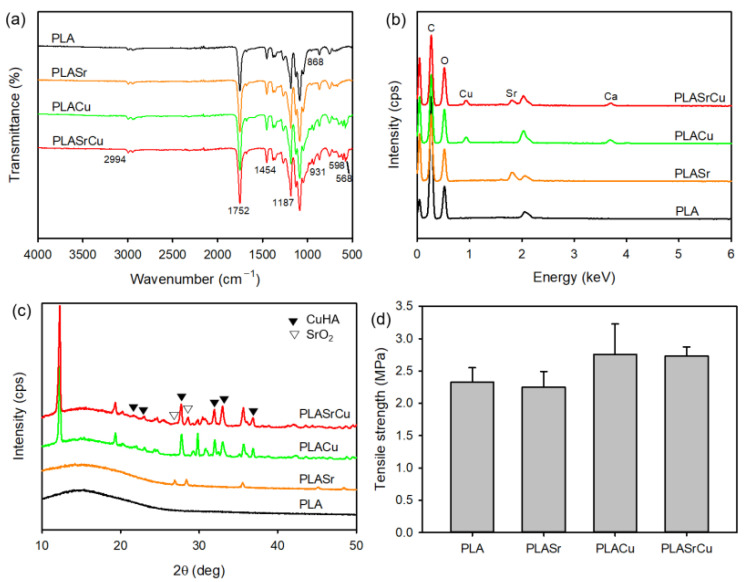
(**a**) ATR–FTIR, (**b**) EDX, (**c**) XRD spectra, and (**d**) tensile strength (*n* = 5) of the composite scaffolds.

ATR–FTIR spectroscopy was used for chemical structure analysis of the PLA nanofibrous composite scaffolds (Figure 4a). The ATR–FTIR spectra of all composite scaffolds exhibited no noticeable changes relative to pristine PLA, meaning that the incorporation of particles did not alter the primary chemical structure of PLA. The absorption peaks at 2994, 1752, and 868 cm^−1^ were assigned to the CH_3_ stretching mode, the C=O stretching mode, and the C–C stretching mode [18,25]. In addition, the characteristic bands at 1454 and 1187 cm^−1^ were assigned to the CH_3_ asymmetric bending mode and the C–O asymmetric stretching mode. Furthermore, new absorption bands at 931, 598, and 568 cm^−1^, corresponding to the P–O stretching mode and the O–P–O bending mode, were observed after the incorporation of CuHA particles [20,25]. In contrast, no significant differences in the ATR–FTIR spectra were detected upon the addition of SrO_2_ particles. The surface elemental composition of the composite scaffolds was further confirmed by EDX (Figure 4b). The pristine PLA scaffold showed characteristic bands corresponding to C and O. The PLASr scaffold showed a new Sr peak originating from the SrO_2_ particles, while the PLACu scaffold displayed an additional Cu peak derived from the CuHA particles. As expected, the PLASrCu scaffold exhibited both Sr and Cu peaks, confirming the successful incorporation of both particles.

The crystalline structure of the PLA nanofibrous composite scaffolds was analyzed via XRD (Figure 4c). The pristine PLA scaffold exhibited broad diffraction peaks, indicative of the amorphous nature of PLA. Upon incorporation of SrO_2_ and/or CuHA particles, distinct diffraction peaks corresponding to the crystalline SrO_2_ and HA crystalline structure were observed. These diffraction patterns were in accordance with the JCPDS database (JCDPDS 01-076-1730 and 09-0432), indicating that Cu doping had minimal influence on the crystal structure of HA. The characteristic peaks at 21.9°, 23.0°, 27.9°, 31.9°, 33.1°, and 37.0° were indexed to the (200), (111), (102), (211), (300), and (310) planes of the HA crystal, respectively [20]. In addition, the diffraction peaks at 26.9°, 28.4°, and 35.5° were due to the (202), (114), and (222) planes of the SrO_2_ crystal [15,26]. For bone tissue regeneration applications, composite scaffolds are required to exhibit adequate mechanical strength to resist physiological loading during bone regeneration. Thus, the mechanical properties of the composite scaffolds were evaluated by tensile testing (Figure 4d). The incorporation of a higher content of particles (PLACu and PLASrCu) resulted in an increased tensile strength, whereas the incorporation of a lower particle content (PLASr) had a negligible effect on tensile strength.

### 3.3. Cell Proliferation on the PLA Nanofibrous Composite Scaffolds

**Figure 5:**
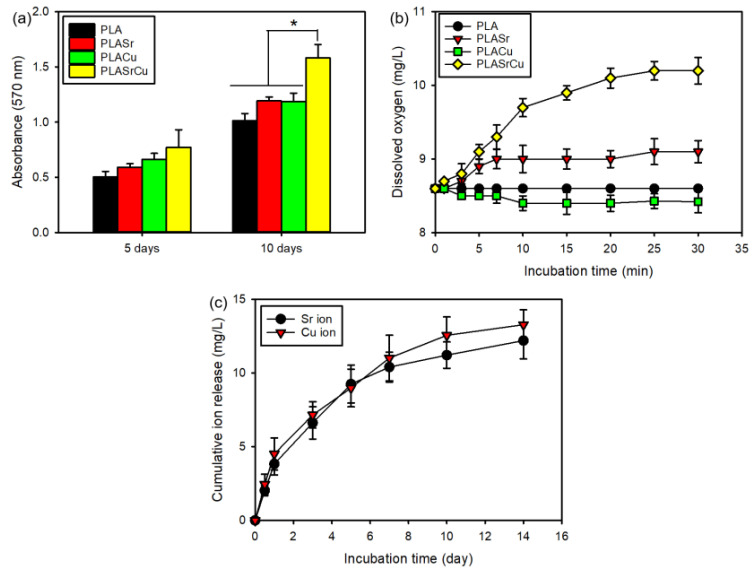
(**a**) Proliferation behavior of MC3T3-E1 cells cultured on the composite scaffolds under hypoxic conditions (*n* = 5). (**b**) Oxygen-generating capacity of the composite scaffolds (*n* = 5). (**c**) Concentrations of Sr and Cu ions as a function of immersion time in DW for the dissolution of the PLASrCu scaffold (*n* = 4). *p* * ˂ 0.05 for the comparison between the two treatment groups.

The biological activity of the PLA nanofibrous composite scaffolds was assessed by evaluating MC3T3-E1 cell proliferation and differentiation. The MTT assay was used to evaluate cell proliferation on the nanofibrous composite scaffolds, with increased cell viability indicating improved biological activity. As expected, MC3T3-E1 cells proliferated more rapidly on the PLASrCu scaffold than on the other scaffolds under hypoxic conditions, suggesting multifunctional effects of SrO_2_ and CuHA incorporation (Figure 5a). Consistently, the superior proliferation observed on the PLASrCu scaffold at all time points can be attributed to the multifunctional effects of sustained Sr and Cu ion release together with oxygen generation [2,5,15,20].

Because angiogenesis remains slow even under conditions where pro-angiogenic signals are present, oxygen-releasing biomaterials are especially critical for sustaining cell viability in osteonecrotic tissue regions. While the PLASr scaffold exhibited limited oxygen generation, the PLASrCu scaffold demonstrated a markedly enhanced and sustained oxygen release profile (Figure 5b). SrO_2_ undergoes hydrolysis to form H_2_O_2_, followed by its decomposition into oxygen and water. In the absence of catalysts, however, H_2_O_2_ decomposes slowly, resulting in delayed oxygen generation [7,10,12]. This limitation was effectively mitigated by Cu ions, which catalyze the decomposition of H_2_O_2_ and enable controlled and sustained oxygen release [27]. Importantly, no initial burst release of oxygen was observed for the PLASrCu scaffold, indicating a stable oxygen-generating behavior for long-term cell survival.

In addition to oxygen generation, the dissolution behavior of microceramics is crucial for promoting osteoblastic proliferation and subsequent mineralization associated with cell layers [10,13,14,15]. The microporous structure of composite scaffolds facilitates water penetration into the scaffold interior, thereby accelerating scaffold decomposition and ion exchange processes. Moreover, the dissolution of microceramics accelerates over time. To further elucidate this behavior, a quantitative analysis of the cumulative Sr and Cu ion release behavior from the PLASrCu scaffold was conducted. The PLASrCu scaffold exhibited an initial rapid and a subsequent sustained release phase, indicating that Sr and Cu ions were maintained within a biologically relevant concentration range (Figure 5c). In addition, the cumulative release of Sr and Cu ions increased steadily over time. According to previous reports, Cu ion concentrations above 10 mg/L exhibit cytotoxicity, whereas lower concentrations showed limited antibacterial activity and failed to achieve the desired antibacterial activity [14,28]. Consistent with these findings, slight cytotoxicity, as evidenced by reduced cell proliferation, was observed in the PLACu scaffold after 10 days of culture (Figure 5a), whereas the PLASrCu did not exhibit any detectable cytotoxicity. Furthermore, when a higher CuHA content (≥50 wt% relative to the PLA weight) was incorporated into the PLASrCu scaffold, significant cytotoxicity was observed, with cell viability decreasing to below 70% relative to the PLASrCu scaffold containing 40 wt% CuHA after 10 days of culture (Figure S1). Based on these results, 40 wt% CuHA relative to the PLA weight was selected as the optimal loading level for the fabrication of the PLACu and PLASrCU scaffolds, providing a favorable balance between cytocompatibility and antibacterial activity. Collectively, these results indicate that the PLASrCu scaffold serves as an efficient bioactive carrier for metal ion and oxygen delivery to promote bone tissue regeneration.

**Figure 6:**
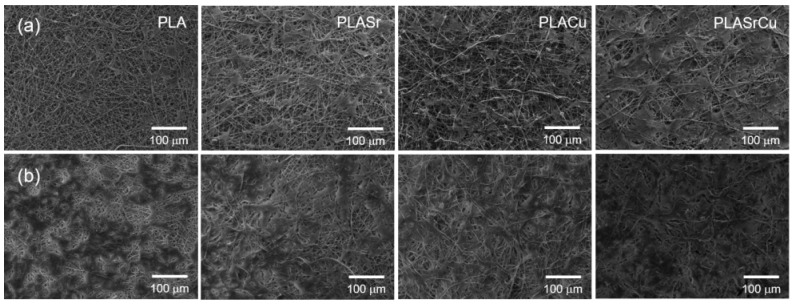
SEM images of MC3T3-E1 cells cultured on the composite scaffolds under hypoxia after (**a**) 5 and (**b**) 10 days of culture.

SEM observations after 5 and 10 days of culture further verified successful MC3T3-E1 cell attachment and spreading on the scaffold surfaces (Figure 6). MC3T3-E1 cells adhered well and exhibited progressive proliferation on all composite scaffolds, indicating favorable biological activity. After 10 days of culture, virtually the whole surface of the PLASrCu scaffold was covered by MC3T3-E1 cells, and this scaffold exhibited the highest cell density.

**Figure 7:**
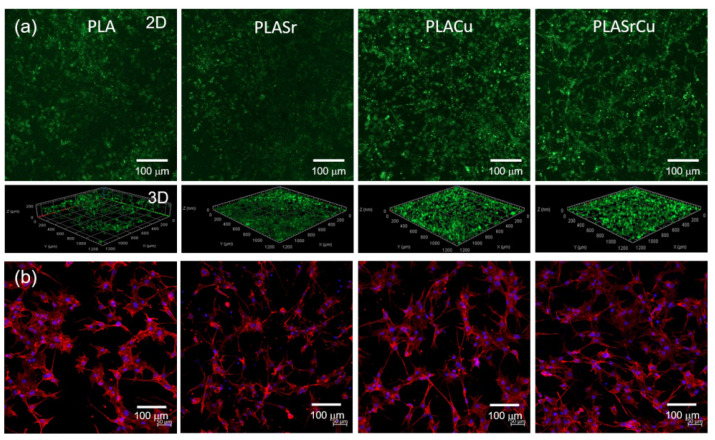
(**a**) Fluorescence microscopy images of live cells stained with calcein-AM on the composite scaffolds and (**b**) CLSM images of MC3T3-E1 cells cultured on the composite scaffolds under hypoxia after culturing for 5 days.

To visually assess the multifunctional biological performance of the PLA nanofibrous composite scaffolds, MC3T3-E1 cells were cultured on the composite scaffolds for 5 days. Cell viability was evaluated through a live-cell staining assay and imaged by confocal laser scanning microscopy (CLSM) (Figure 7a). Live cells exhibited green fluorescence following calcein-AM staining. MC3T3-E1 cells proliferated successfully on all composite scaffolds, with a markedly higher number of stained cells observed on the PLASrCu scaffold, in agreement with the results of MTT assay.

To further examine cytoskeletal organization, CLSM imaging was conducted after 5 days of culture, followed by phalloidin-TRITC (red) staining for F-actin and DAPI (blue) counterstaining for nuclei. MC3T3-E1 cells displayed strong adhesion to all composite scaffolds and exhibited a typical spindle-shaped morphology (Figure 7b). Well-organized and elongated actin filaments were evenly dispersed over the scaffold surfaces. Notably, the PLASrCu scaffold supported a substantially higher number of attached cells compared with the other composite scaffolds.

### 3.4. Cell Differentiation and Mineralization on the PLA Nanofibrous Composite Scaffolds

**Figure 8:**
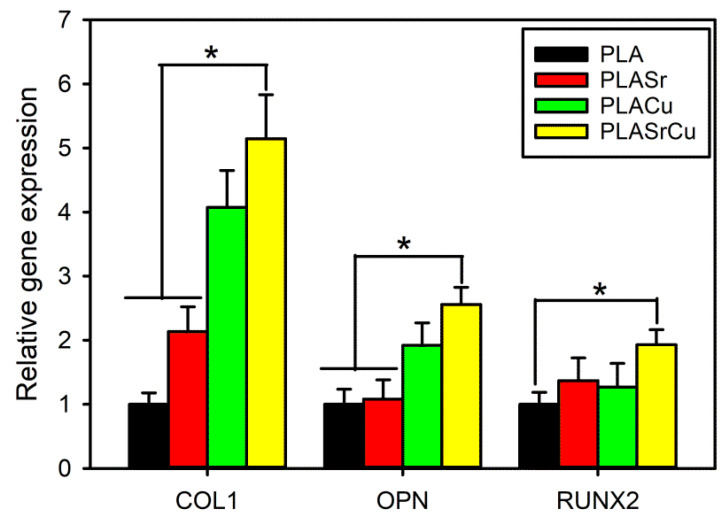
Gene expressions in osteogenic differentiation (COL1, OPN, and RUNX2) of MC3T3-E1 cells cultured on the composite scaffolds for 14 days (*n* = 5). *p* * ˂ 0.05 for the comparison between the two treatment groups.

Osteogenic gene expression analysis further verified the excellent biological activity of the PLASrCu scaffold. The mRNA expression levels of late-stage osteogenic markers (COL1, OPN, and RUNX2) were evaluated using RT-qPCR. As the primary ECM protein in bone tissue, COL 1 is essential for bone remodeling [29]. OPN plays a role in bone formation and repair, whereas RUNX2 functions as a key transcriptional regulator of osteoblast differentiation. MC3T3-E1 cells grown on the PLASrCu scaffold exhibited markedly elevated expression of these genes relative to cells cultured on the other scaffolds (Figure 8), demonstrating a strong stimulatory effect on osteoblastic differentiation. These results are consistent with previous studies reporting that Sr- and Cu-doped microceramics enhance osteogenesis by promoting bone formation while inhibiting bone resorption [10,13,14,15]. Taken together, these results indicate that the incorporation of SrO_2_ and CuHA particles modulates both initial cellular response, such as cell adhesion and proliferation, and subsequent long-term osteogenic differentiation.

**Figure 9:**
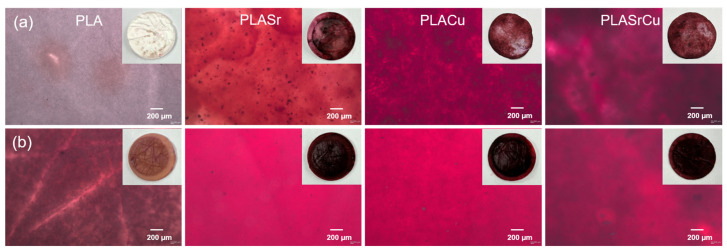
ARS staining of MC3T3-E1 cells cultured on the composite scaffolds under hypoxia (**a**) before cell seeding and (**b**) after culturing for 14 days. Inserts are digital images of the same samples.

The deposition of calcium nodules on the composite scaffolds is widely used as a marker of late-stage osteogenic differentiation and mineralization [2,14]. ARS selectively reacts with calcium salts, producing orange-red precipitates whose intensity and spatial distribution correspond to the degree of bone-like mineral formation. Prior to cell seeding, all composite scaffolds except the pristine PLA scaffold exhibited a slight orange-red coloration due to the interaction of ARS with Sr- and Ca-containing components (Figure 9a). After 14 days of culture, calcium nodule formation was markedly increased on all composite scaffolds (Figure 9b). Notably, the PLASrCu scaffold exhibited deeper and more extensive orange-red staining compared with the other scaffolds, indicating significantly enhanced mineralization of MC3T3-E1 cells. Overall, these results demonstrate that the PLASrCu scaffold possesses superior capability to promote osteoblastic differentiation and mineral deposition, highlighting its strong potential for enhancing biological activity and supporting bone tissue regeneration.

### 3.5. Antibacterial Activity of PLA Nanofibrous Composite Scaffolds

**Figure 10:**
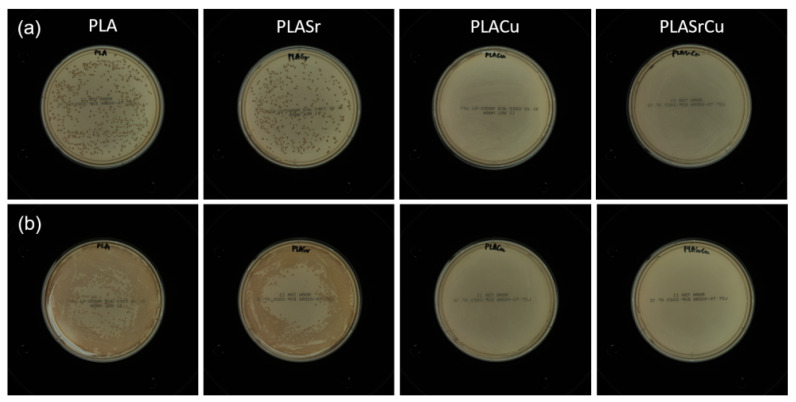
Optical images of (**a**) *S. aureus* and (**b**) *P. aeruginosa* colonies after incubation with the composite scaffolds.

The prevention of postoperative infections remains a major challenge in developing bone-regenerative materials. To evaluate the antibacterial activity of the PLA nanofibrous composite scaffolds against *S. aureus* and *P. aeruginosa*, a dilution agar plate method was employed [20]. The surfaces of the PLA and PLASr scaffolds exhibited substantial bacterial adhesion of both *S. aureus* and *P. aeruginosa*, indicating poor intrinsic antibacterial properties (Figure 10). In contrast, the PLA composite scaffolds containing the CuHA particles (PLACu and PLASrCu) demonstrated markedly enhanced antibacterial activities against both bacterial strains, as evidenced by a significant reduction in bacterial colony formation. The antibacterial efficiencies of the PLACu and PLASrCu scaffolds against *S. aureus* and *P. aeruginosa* exceeded 99.9% compared with the pristine PLA scaffold, which can be attributed to the antibacterial effects of Cu ions [14].

The antibacterial efficacy of Cu ions arises from multiple mechanisms, including disruption of bacterial cell membranes, inhibition of essential metabolic pathways, and production of ROS, thereby providing broad-spectrum antimicrobial activity [14]. Concurrently, Cu ions play a crucial role in bone regeneration, serving as cofactors for key enzymes and cytokines and thus enhancing osteoblastic proliferation and differentiation. Collectively, the incorporation of SrO_2_ and CuHA particles significantly improved the osteoconductive, osteoinductive, and antibacterial performance of the PLASrCu scaffold, making it a potential multifunctional platform for enhanced bone regeneration.

## 4. Conclusions

In this study, a multifunctional PLA nanofibrous composite scaffold incorporating SrO_2_ and CuHA particles was successfully developed for bone tissue regeneration. The PLASrCu scaffold exhibited an interconnected ECM-like architecture with preserved chemical structure and enhanced mechanical properties. Sustained release of Sr and Cu ions, together with controlled oxygen generation, significantly promoted osteoblast adhesion, proliferation, and survival under hypoxic conditions. Importantly, Cu ions efficiently catalyzed H_2_O_2_ decomposition, enabling stable oxygen release without cytotoxic burst effects. The PLASrCu scaffold markedly enhanced osteogenic differentiation and mineralization, as confirmed by upregulated osteogenic gene expression and calcium deposition. In addition, strong antibacterial activity against both *S. aureus* and *P. aeruginosa* was achieved due to the presence of Cu ions. Overall, these enhanced bioactive, oxygen-generating, and antibacterial properties establish the PLASrCu scaffold as a promising platform for treating infected and oxygen-deficient bone defects.

## Data Availability

The original contributions presented in this study are included in the article. Further inquiries can be directed to the corresponding author.

## Supplementary Materials

The following supporting information can be downloaded at https://www.mdpi.com/article/10.3390/ma19142982/s1, Figure S1: Relative viability of MC3T3-E1 cells cultured on PLASrCu scaffolds containing different CuHA contents after 10 days of culture.
