# Supplementary material for: Effect of Strontium Peroxide and Copper-Doped Hydroxyapatite Microceramics on the Osteogenesis and Antibacterial Activity of Nanofibrous Composite Scaffolds
Source: Materials (Basel). 2026 Jul 10;19(14):2982. doi: 10.3390/ma19142982 (PMC13412465; doi:10.3390/ma19142982)
Supplement: Supplementary file 1 [file materials-19-02982-s001.zip › materials-4394790-supplementary.pdf]

Supplementary Materials

Effect of Strontium Peroxide and Copper-Doped Hydroxyapatite Microceramics on the Osteogenesis and Antibacterial Activity of Nanofibrous Composite Scaffolds

Pan-Geon Park [1] and Young-Jin Kim [1,*]

[1] Department of Advanced Materials and Chemical Engineering, Daegu Catholic University, Gyeongsan 38430, Republic of Korea; psb7193@naver.com

* Correspondence: yjkim@cu.ac.kr; Tel.: +82-53-850-2512

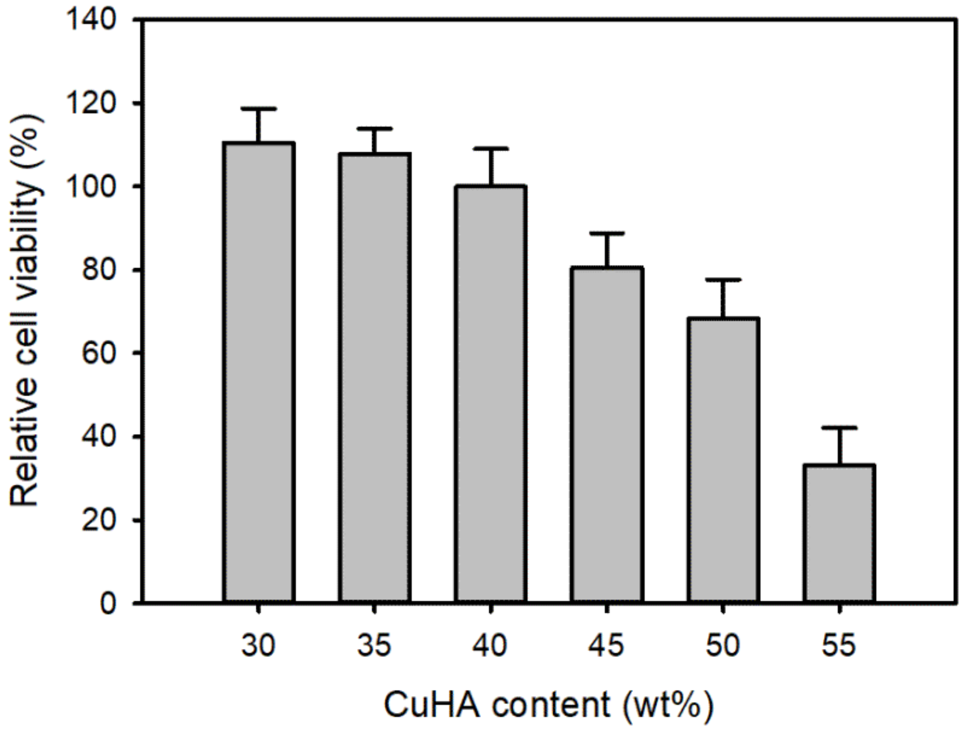

Figure S1. Relative viability of MC3T3-E1 cells cultured on PLASrCu scaffolds containing different CuHA contents after 10 days of culture
